# Assessment of the Abundance and Potential Function of Human Papillomavirus Type 16 Circular E7 RNA

**DOI:** 10.1128/mbio.00411-22

**Published:** 2022-05-17

**Authors:** Richard C. Wang, Eunice E. Lee, Jiawei Zhao, Jiwoong Kim

**Affiliations:** a Department of Dermatology, UT Southwestern Medical Center, Dallas, Texas, USA; b Simmons Comprehensive Cancer Center, UT Southwestern Medical Center, Dallas, Texas, USA; c Division of Hematology/Oncology, Boston Children’s Hospital, Harvard Medical School, Boston, Massachusetts, USA; d Quantitative Biomedical Research Center, UT Southwestern Medical Center, Dallas, Texas, USA; Virginia Polytechnic Institute and State University

**Keywords:** RNA splicing, circular RNA, oncogenic virus, papillomavirus, translation

## LETTER

We read with interest Yu and Zheng’s article, “Human Papillomavirus Type 16 Circular RNA Is Barely Detectable for the Claimed Biological Activity” (1). In that article, the authors confirm previously reported findings that human papillomaviruses (HPVs) generate circular E7 (circE7) RNA (2). Yu and Zheng confirmed the presence of HPV16 circE7 as an RNase R-resistant circular RNA (circRNA), with the previously reported back-splice junction, in CaSki cells. They also detect it in HPV16-positive cervical epithelial cells (W12 subclonal lines) (1). However, the authors then question whether circE7 is biologically functional. Using only digital droplet reverse transcription-PCR (RT-PCR), the authors contend that circE7 is present at less than 1 copy per cell. Next, using small interfering RNA (siRNA) alone, the authors argue that previously reported phenotypes attributed to circE7 were the result of off-target effects on HPV16 E6*I RNA. In addition, the authors misstate the main conclusions of the original article describing circE7. Thus, we would like to comment on the abundance and potential functions of circE7 and clarify any misunderstandings regarding the well-established roles for E6*I linear RNA in E7 protein production.

**FIG 1:**
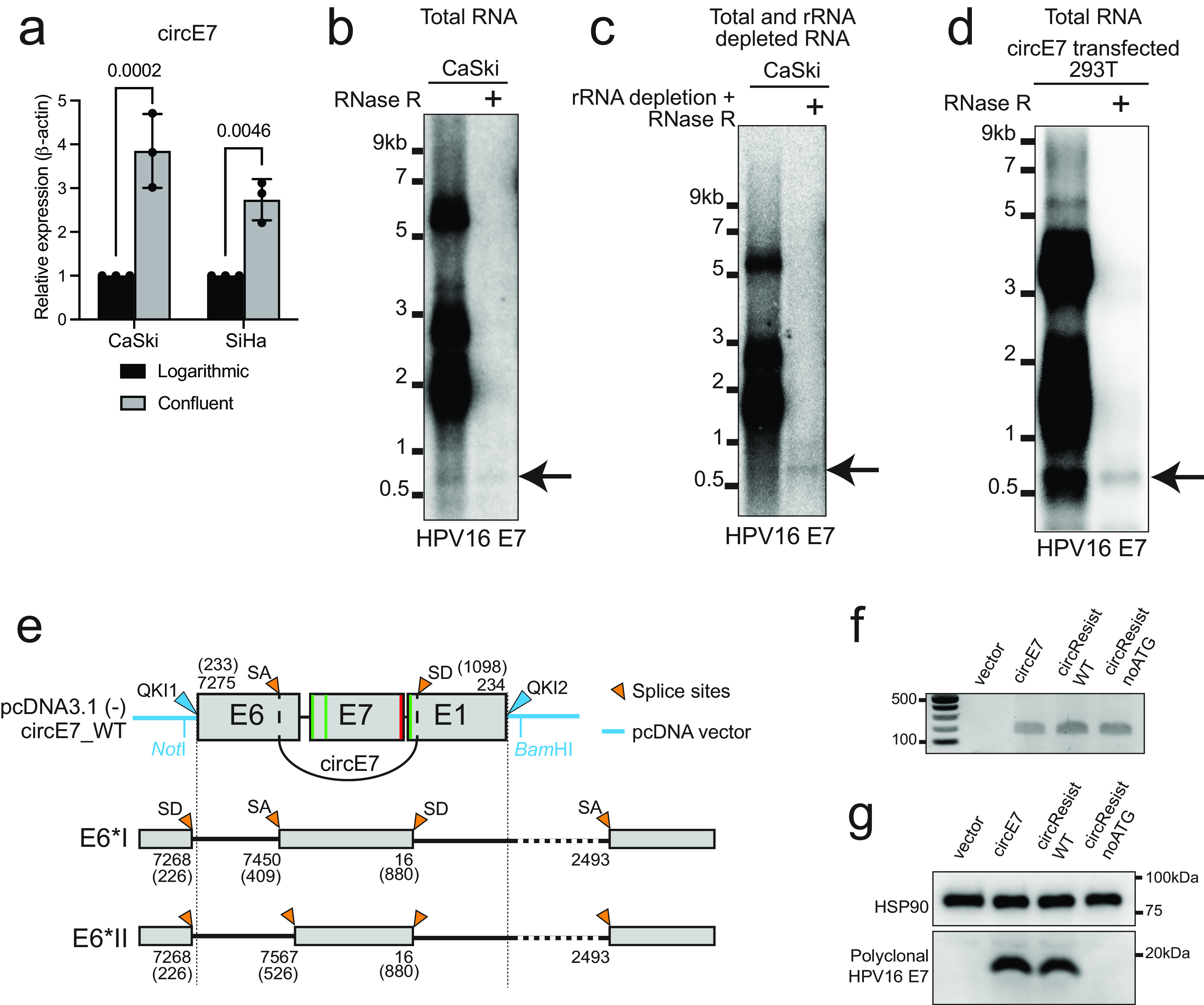
Characterization of circE7 by RT-PCR, Northern blotting, and lentiviral expression. (a) CaSki and SiHa cells were cultured to ~50% density (logarithmic) or near confluence. Relative levels of circE7 were assessed by qRT-PCR (normalized to β-actin). Expression levels in confluent cells are presented relative to a logarithmic culture prepared in parallel (*n* = 3 biological replicates). Data are shown as means ± standard deviations. *P* values were calculated by analysis of variance (ANOVA) with Sidak correction. (b) Northern blotting of total RNA (15 μg per lane) with RNase R treatment (20 U for 60 min) for the indicated lane. Arrows indicate RNase R-resistant bands. Quantitation indicates that circE7 accounts for 1.7% of the total E7 signal in this blot. The Northern blot was probed with an E7 probe generated by random-hexamer primer labeling of a 450-bp fragment of HPV16 E7. (c) Northern blotting of total (10 μg for the untreated lane) or rRNA-depleted, RNase R-treated RNA (20 μg total RNA treated by NEBNext rRNA depletion, followed by RNase R at 10 U for 30 min). The Northern blot was probed with HPV16 E7. (d) Northern blotting of 293T cells transfected with the circE7 expression construct with RNase R treatment (20 U for 30 min) for the indicated lane. The Northern blot was probed with HPV16 E7. (e) Map of the cloned circE7 region demonstrating the absence of upstream splice donor (SD) sequences for E6*I and E6*II in circE7 constructs. HPV genomic positions are labeled according to the NCBI HPV16 reference genome. Conventional genomic positions are indicated in parentheses. SA, splice acceptor. (f) RT-PCR confirms the formation of circE7 RNAs from circE7 lentivirally transduced BJ-hTERT fibroblasts. Total RNA (2 μg) was treated with 5 U of RNase R for 40 min prior to a random-hexamer-primed RT reaction for all cell lines. (g) Western blotting (WB) confirms that circE7 and circResist_WT, but not circResist_noATG, generate E7 protein in lentivirally transduced BJ-hTERT cells. HSP90 is a loading control.

One contention raised by Yu and Zheng is that circE7 is not present at a high enough level to be biologically significant. Low abundance does not preclude an RNA from being biologically functional. Indeed, there are many examples of biologically functional RNAs that are present at low copy numbers (3, 4). More importantly, we have several lines of evidence that circE7 is more abundant than suggested by Yu and Zheng. We previously reported that *in vitro* growth conditions can influence circE7 levels (2). Notably, we have found that cell density can impact the levels of circE7. CaSki and SiHa cells grown to a high density had 2- to 4-fold more circE7 than nonconfluent cells (Fig. 1a). Thus, cell density and other differences in *in vitro* growth conditions may help to explain the low levels of circE7 reported by Yu and Zheng.

Differences in the methods of detection could explain the apparent differences in the levels of circE7. Yu and Zheng quantitated the levels of circE7 using a single methodology, digital droplet PCR after reverse transcription. Digital PCR (dPCR) is prone to underestimation bias (5, 6). RT-dPCR is especially susceptible to an underestimation of target abundance through “molecular dropout” artifacts, especially when templates contain secondary structures (7). Notably, the HPV16 circE7 RNA region is predicted to form an internal ribosome entry site (IRES)-like hairpin involved in binding the 18S ribosome (8), likely making RT and PCR amplification of circE7 more prone to dropout artifacts. In our quantitative RT-PCR (qRT-PCR) experiments, we found that circE7 was ~0.2 to 1% of the E6*I transcripts, rather than the ~0.06% estimated by Yu and Zheng. While different primers and different RT and amplification conditions could explain the variability in the levels of circE7 detected, these differences also highlight the limitations of using RT-PCR alone to estimate transcript abundance.

In contrast to RT-PCR, Northern blot assays are more reliable and not liable to detection artifacts. In our study, we quantitated Northern blots of total RNA from CaSki, SiHa, and SCC154 cells using an E7 probe generated by random-hexamer-primed labeling of a 450-bp fragment of E7. This fragment was amplified by PCR from a plasmid containing the full-length HPV16 genome. An RNase R-resistant E7 band was identified in multiple independent experiments. circE7 consistently accounted for ~1 to 3% of E7-containing transcripts, or ~7 to 21 copies/cell (Fig. 1b). Our Northern blot assays examined total RNA, rather than poly(A)-enriched RNA, which may explain some of the differences in cell fractionation data from previous publications (9). Northern blot assays using rRNA-depleted RNA also identified the same RNase R-resistant circE7 band (Fig. 1c). The identity of the band as circE7 is further confirmed by the fact that an RNase R-resistant band of the same size is generated by 293T cells transfected with a circE7 expression plasmid (Fig. 1d). Because the circE7 expression construct lacks the upstream sequences required to generate E6*I or other known linear E7 mRNAs (Fig. 1e), these transfection experiments confirm the identity of the band present in Northern blots as circE7. Yu and Zheng express concerns about the approximate size of the circE7 band, but it is well established that circular RNAs migrate more slowly than linear RNAs (10–13).

The abundance of circE7 is further bolstered by data from RNA sequencing, which is also less sensitive to artifacts than RT-dPCR. Publicly available Sequence Read Archive (SRA) data and The Cancer Genome Atlas (TCGA) sequencing data demonstrate that circE7 is present in both cervical cancer and head and neck squamous cell carcinoma (HNSCC). More recent analyses have demonstrated circE7 to be present in anal squamous cell cancer (14) and vulvar squamous cell cancer (15). The proportion of back-spliced reads corresponding to circE7 varied between samples. However, in many samples, circE7 back-splice-specific reads represented >5% of all reads covering that splice junction (2). RT-PCR, Northern blot, and RNA sequencing results all confirm the presence of circE7 as a biologically relevant proportion of spliced RNA from the HPV early region. Our findings also highlight the importance of using orthogonal methodologies to verify the identity and abundance of putative circRNAs (16).

Another point raised by the authors was related to the specificity of the RNA interference (RNAi) used to assess circE7’s function. Using siRNAs, Yu and Zheng were unsuccessful in their attempts to knock down circE7 without also depleting E6*I RNA. In contrast, we employed a lentiviral, doxycycline-inducible short hairpin RNA (shRNA) vector to knock down circE7 and were successful in knocking down circE7 without significantly affecting E6*I levels as assessed by both RT-PCR and Northern blotting (2). Compared to lentivirus-mediated shRNAs, siRNAs have a great propensity for inducing off-target effects (17, 18). Also, different sequences were used to conduct the RNAi studies. Thus, differences in RNAi methodologies and protein detection protocols might help to explain why the siRNA transfection experiments presented by Yu and Zheng showed less specificity.

An important control for the specificity of our shRNA knockdown experiments was the rescue of the shRNA knockdown through the expression of an shRNA-resistant circE7 (2). Lentiviral constructs expressing either an shRNA-resistant wild-type (WT) circE7 (circResist_WT) or a mutant shRNA-resistant circE7 in which potential E7 start codons have been mutated (circResist_noATG) were generated. The lentiviral circE7 expression constructs used to express circE7 do not possess the upstream HPV early sequences required to generate E6*I, E6*II, or other known linear E7-encoding transcripts (Fig. 1e). Thus, E7 oncoprotein expression from the minigene is derived from the circE7 RNA. When cotransduced with the circE7 shRNA, both shRNA-resistant circE7 plasmids were able to rescue circE7 RNA expression as assessed by qRT-PCR (2). However, only circResist_WT, but not circResist_noATG, rescued E7 protein expression and cell proliferation (2). We have confirmed the expected functions of these lentiviral circE7 expression constructs in BJ dermal fibroblasts, which do not contain HPV sequences. Specifically, WT circE7, circResist_WT, and circResist_noATG each generated the expected circE7 RNAs as demonstrated by the detection of RNase R-resistant inverse RT-PCR products (Fig. 1f). However, only the circE7 and circResist_WT constructs generated E7 protein (Fig. 1g). Thus, the lentiviral WT circE7 constructs, which cannot generate E6*I or any other known spliced linear E7 RNA but do generate abundant circE7, produce the E7 protein. Thus, we provide evidence that circE7 is biologically functional and translated into the E7 oncoprotein.

Finally, the authors inaccurately state that we conclude that “E6*I is nuclear and unable to encode E7” (1). This is obviously not true as many groups have demonstrated that most E7 protein is derived from linear E6*I RNA, a fact that is acknowledged multiple times in our original publication (2, 19–22). Our discoveries do not dispute the role of E6*I in E7 oncoprotein production but rather build upon these important studies and demonstrate that previously unrecognized circular E7 isoforms are also required for optimal E7 protein production. The biochemical characterization of circE7 demonstrates that it possesses features of a translated circRNA (23). It is localized to the cytoplasm, N^6^-methyladenosine modified, and associated with polysomes. In addition, circE7-mediated E7 expression is induced by heat shock. These biochemical features are consistent with overexpression and knockdown studies that demonstrate that circE7 functions as a translated circRNA. Additional studies will be required to determine whether linear and circular E7 isoforms interact cooperatively or independently to promote E7 production in viral replication and tumorigenesis.
